# Survival Data Analysis with Time-Dependent Covariates Using Generalized Additive Models

**DOI:** 10.1155/2012/986176

**Published:** 2012-04-01

**Authors:** Masaaki Tsujitani, Yusuke Tanaka, Masato Sakon

**Affiliations:** ^1^Department of Engineering Informatics, Osaka Electro-Communication University, Osaka 572-8530, Japan; ^2^Clinical Information Division, Data Science Center, EPS Corporation, Osaka 532-0003, Japan; ^3^Nishinomiya Municipal Central Hospital, Hyogo 663-8014, Japan

## Abstract

We discuss a flexible method for modeling survival data using penalized smoothing splines when the values of covariates change for the duration of the study. The Cox proportional hazards model has been widely used for the analysis of treatment and prognostic effects with censored survival data. However, a number of theoretical problems with respect to the baseline survival function remain unsolved. We use the generalized additive models (GAMs) with B splines to estimate the survival function and select the optimum smoothing parameters based on a variant multifold cross-validation (CV) method. The methods are compared with the generalized cross-validation (GCV) method using data from a long-term study of patients with primary biliary cirrhosis (PBC).

## 1. Introduction

Several prognostic models for PBC data have been developed using the Cox proportional hazards model, and the values of all covariates were determined at the time when the patient entered the study [1]. However, situations may exist in which the values of covariates change for the duration of the study. The time-dependent model uses follow-up data to estimate the effect of the evolution of the covariates during the course of the disease; see, for example, Cox [2], Altman and Stavola [3] and Collett [4].

Let *t* be a continuous lifetime variable and **x** = (*x*_1_,…, *x*_*I*_) a vector of *time-fixed* covariates. The Cox's proportional hazards model postulates that the hazard at time *t* is the product of two components [5, 6]

(1) $$h\left(t;x\right)=h_{0}\left(t\right)\exp\left[\overset{I}{\underset{i=1}{\sum }}b_{i}x_{i}\right],$$

where **b** = (*b*_1_,…, *b*_*I*_) is a vector of coefficients. The proportional hazards assumption is that the baseline hazard *h*_0_(*t*) is a function of *t* but does not involve the values of covariates **x** which are measured at the beginning of an interval to predict short-term survival.

We investigate PBC data for 312 patients who were seen at the Mayo Clinic and were monitored for the duration of the study, as described in Murtaugh et al. [7] and Therneau and Grambsch [8]. The Cox proportional hazards model was developed based on the relationship between survival and the patient characteristics observed when the patient entered the study. The precision of time-fixed models used in PBC is rather low, partly because these models are based on data for which the covariates were measured at the time when the patient entered the study.

For the analysis of data with time-dependent covariates, however, the survivor function for any individual depends on time *t* and the baseline hazard function. This means that the survivor function cannot be expressed as a power of the baseline survivor function and is generally difficult to obtain for any individual; see, for example, Kalbfleish and Prentice [9] and Marubini and Valsecchi [10]. The Mayo updated model (e.g., [7]), and the European new version model (e.g., [3, 11, 12]) have been commonly used to improve the accuracy of survival predictions as a function of covariates measured at any time during the course of the disease. In the present article, we propose the variant multifold CV method for GAM when choosing the optimum smoothing parameters in order to estimate the survival function and predict the short-term survival (say, for the following six months) at any time during the course of the disease.

Another useful idea in our analysis is the concept of competing risk. There is “liver transplantation” in PBC data as competing risk. Competing risk has been treated as censored data. By adding the liver transplantation as one of time-dependent covariate, one can test the significance of liver transplantation.

## 2. Model Building

By extending the Cox proportional hazard model (1), a flexible survival model has been examined

(2) $$h\left(t;x\right)=h_{0}\left(t\right)\exp\left[\overset{I}{\underset{i=1}{\sum }}s_{i}\left(x_{i}\right)\right],$$

where *s*_*i*_(**x**_*i*_) is a spline function for the covariate **x**_*i*_ [13–15]. The proportional hazard model (2) used the time-fixed values of covariates as shown in Dickson et al. [1]. The estimates of hazard ratio by relative survival regression model [16] with time-dependent covariates are compared with that of Cox proportional hazard model. A new approach [17, 18] is proposed with PBC data, aiming to capture nonlinear patterns of bilirubin time courses and their relationship with survival time of patients. However, because most patients with PBC make repeated visits to the clinic, it is natural to ask the optimum timing of liver transplantation by predicting short-term survival at any time in the course of the disease.

The time-dependent covariates **X**_*l*_^〈*d*〉^ = (*a*_*l*_^〈*d*〉^, *x*_*l*1_^〈*d*〉^,…, *x*_*lI*_^〈*d*〉^) are provided for patient #*d*, where *a*_*l*_^〈*d*〉^ is the midpoint of time interval [*t*_*l*_^〈*d*〉^, *t*_*l*+1_^〈*d*〉^] for the *l*th clinic visit. The event times may be subject to the usual random censoring. Then only the minimum of survival and censoring time with censoring indicator

(3) $$\delta_{l}^{\langle d\rangle }=\{\begin{array}{cc} 1:\text{patient}\;\#d\;\text{died}\;\text{at}\;\text{time-interval} & \\ \;\text{for}\;\text{the}\;l\text{th}\;\text{clinic}\;\text{visit} & \\ 0:\text{ otherwise} & \end{array}$$

are observed. The relative hazard *h*(*t*)/*h*_0_(*t*) then depends on time *t*, and thus the proportional hazards assumption is no longer satisfied, as described in Altman and Stavola [3] and Arjas [19].

For example, Table 1 shows the values of *age*, *prothrombin time*, and *bilirubin* as time-dependent covariates for dead patient #9; for details, see Table 4 in Murtaugh et al. [7]. Patients were scheduled to return for further observations at six months, 12 months, and yearly. Thus, *n* = 312 patients generate 1945 observations in total. The covariates values for each patient were allowed to vary with the time interval for the *l*th clinic visit.

A grouped version of Cox's proportional hazard model with time-fixed covariates has been considered in the framework of discrete grouped data for the feed-forward neural network. Given the continuous survivor time, piecewise models arise from the partition of the time axis into disjointed intervals. Biganzoli et al. [20, 21] show that, by treating the time interval as an input variable in a feed forward neural network, it is possible to estimate smoothed discrete hazards as conditional probabilities of failure. Biganzoli et al. [20] also pointed out that an advantage of this kind of data structure is the possibility of straightforward use of time-dependent covariates since each subject is represented, for each observation interval, by one input vector which can change across intervals. In order to apply this neural network approach, which is called partial logistic regression models [20], discretization of one-month or one-week intervals must be applied for the continuous survivor time with time-fixed covariates. We cannot determine which discretization, one-month or one-week intervals, must be applied; that, is the discretization is not originally unique. For the data in Table 1, however, the choice of discretization of the time axis for the partial logistic regression model is generally determined by clinical relevance, possibly according to the scheduled time intervals between follow-up visits.

The primary goal of the present study is to predict short-term survival in patients on the basis of measurements of several characteristics having time-dependent covariates **X**_*l*_^〈*d*〉^ for the purpose of facilitating the decision as to when to undertake liver transplantation. Based on partial logistic model due to Cox [22] and Efron [23] for the grouped data, Tsujitani and Sakon [24] have proposed a partial logistic model with a discrete hazard rate *h*_*l*_^<*d*>^ for ungrouped data having time-dependent covariates

(4) $$\begin{array}{cc} \ln\left(\frac{h_{l}^{\langle d\rangle }}{1-h_{l}^{\langle d\rangle }}\right)=\beta_{0}+\gamma t_{l}^{\langle d\rangle }+\beta_{1}x_{l1}^{\langle d\rangle }+\beta_{2}x_{l2}^{\langle d\rangle }+\cdots +\beta_{I}x_{lI}^{\langle d\rangle }, & \\ d=1,2,\ldots .,n, & \end{array}$$

where **β** = (*β*_1_,…, *β*_*I*_) is a vector of coefficients. The modeled response is the logit of hazard rate, and the logit is linear in the covariates. However, this assumption is violated when covariate effects are best represented by smooth, nonlinear function. In recent years, a variety of powerful techniques have been developed for exploring the function form of effects. We examine here a flexible survival model GAM that does not require linearity of the covariate function by extending a generalized linear model (GLM); see, for example, Hastie and Tibshirani [13] and McCullagh and Nelder [25]. By identification of nonlinear covariate effects, we can estimate more accurately a patient's prognosis and thus determine a liver transplant based on prediction of short-term survival.

The linear predictor in (4) is specified as a sum of smooth functions *s*(**x**) with twice continuous derivatives of some or all of the covariates for the discrete hazard rate *h*_*l*_^<*d*>^ of patient #*d* at the time interval *l*

(5) $$\begin{array}{cc} \ln\left(\frac{h_{l}^{\langle d\rangle }}{1-h_{l}^{\langle d\rangle }}\right) & =\beta_{0}+s_{0}\left(t_{l}^{\langle d\rangle }\right)+s_{1}\left(x_{l1}^{\langle d\rangle }\right)\\ & \;+s_{2}\left(x_{l2}^{\langle d\rangle }\right)+\cdots +s_{I}\left(x_{lI}^{\langle d\rangle }\right). \end{array}$$

The smooth functions in (5) can be represented as

(6) $$\begin{array}{cc} s_{0}\left(x\right) & =\overset{q_{0}}{\underset{j=1}{\sum }}\beta_{j}b_{0j}\left(t\right),\\ \;s_{1}\left(x\right) & =\overset{q_{1}}{\underset{j=1}{\sum }}\beta_{q_{0}+j}b_{1j}\left(x\right),\\ \ldots & \\ s_{I}\left(x\right) & =\overset{q_{I}}{\underset{j=1}{\sum }}\beta_{q_{I-1}+j}b_{Ij}\left(x\right), \end{array}$$

where *q*_1_, *q*_2_,…, *q*_*I*_ are the numbers of knots, and

(7) $$\begin{array}{cc} \beta & =(\beta_{0},\beta_{1},\ldots ,\beta_{q_{0}},\beta_{q_{0}+1}{,\beta }_{q_{0}+2},\ldots ,\beta_{q_{0}+q_{1}},\\ & \;\;\ldots ,\beta_{q_{0}+q_{1}+\cdots +q_{I-1}+1}{,\beta }_{q_{0}+q_{1}+\cdots +q_{I-1}+2},\\ & \;\ldots ,\beta_{q_{0}+q_{1}+\cdots +q_{I}}), \end{array}$$

(8) $$\begin{array}{c} \begin{array}{cc} Z & =\left[\begin{array}{cccccccc} 1 & b_{01}\left(x_{11}\right) & \ldots & b_{0q_{1}}\left(x_{11}\right) & \cdot & b_{I-1,q_{0}+q_{1}+\cdots +q_{I-1}+1}\left(x_{I-1,1}\right) & \ldots & b_{I,q_{0}+q_{1}+\cdots +q_{I}}\left(x_{I1}\right)\\ 1 & b_{01}\left(x_{12}\right) & \ldots & b_{0q_{1}}\left(x_{12}\right) & \cdot & b_{I-1,q_{0}+q_{1}+\cdots +q_{I-1}+1}\left(x_{I-1,2}\right) & \ldots & b_{I,q_{0}+q_{1}+\cdots +q_{I}}\left(x_{I2}\right)\\ \cdot & \cdot & \ldots & \cdot & \cdot & \ldots & \ldots & \cdot \\ 1 & b_{01}\left(x_{11}\right) & \ldots & b_{0q_{1}}\left(x_{1n}\right) & \cdot & b_{I-1,q_{0}+q_{1}+\cdots +q_{I-1}+1}\left(x_{I-1,n}\right) & \ldots & b_{I,q_{0}+q_{1}+\cdots +q_{I}}\left(x_{In}\right) \end{array}\right] \end{array}. \end{array}$$

The functions *b*_*ij*_(*x*_*ij*_) in the matrix **Z** are *B*-spline basis functions. Thus, (5) can be rewritten as

(9) $$\ln\left(\frac{h_{l}^{\langle d\rangle }}{1-h_{l}^{\langle d\rangle }}\right)=\underset{n\times q\;q\times 1}{Z\;\beta };\;q=\overset{I}{\underset{i=0}{\sum }}q_{i},$$

where *B*-splines with 10 interiors knots will be used for each continuous covariate. The number of knots is arbitrary but appears to have little effect on the results, provided that the number is not too small, as described in Gray [15].

At the time interval for the *l*th clinic visit of patient #*d*, we define

(10) $$\begin{array}{cc} \delta_{l}^{\prime \langle d\rangle } & =\{\begin{array}{cc} 1:\text{patient}\;\#d\;\text{was}\;\text{censored}\;\text{at} & \\ \text{ time-interval for}\;\text{the}\;l\text{th}\;\text{clinic}\;\text{visit} & \\ 0:\text{ otherwise}, & \end{array}\\ v_{l}^{\langle d\rangle } & =\left(\delta_{1}^{\langle d\rangle },\delta_{1}^{\prime \langle d\rangle },\delta_{2}^{\langle d\rangle },\delta_{2}^{\prime \langle d\rangle },\ldots ,\delta_{l-1}^{\langle d\rangle }{,\delta }_{l-1}^{\prime \langle d\rangle }\right)\\ & =\left(0,0,\ldots ,0\right),\\ v_{l}^{\prime \langle d\rangle } & =\left(v_{l}^{\langle d\rangle }{,\delta }_{l}^{\langle d\rangle }\right)=\left(0,0,\ldots ,0,\delta_{l}^{\langle d\rangle }\right), \end{array}$$

where **v**_*l*_^〈*d*〉^ is the history of dead and censored of time intervals for the first *l*th clinic visit of patient #*d*, and **v**_*l*_^′〈*d*〉^ = (**v**_*l*_^〈*d*〉^, *δ*_*l*_^〈*d*〉^) is the same history extended to include *δ*_*l*_^<*d*>^. Tsujitani and Sakon [24] derived the full log likelihood for all patients

(11) $$\begin{array}{c} \ln L=\ln L\left(\beta \right)+\overset{n}{\underset{d=1}{\sum }}\;\overset{l_{d}}{\underset{l=1}{\sum }}\ln p\left(\delta_{l}^{\prime \langle d\rangle }\;|\;v_{l}^{\prime \langle d\rangle }\right) \end{array}$$

with partial log likelihood

(12) $$\begin{array}{cc} \ln L\left(\beta \right) & =\overset{n}{\underset{d=1}{\sum }}\{\overset{l_{d}-1}{\underset{l=1}{\sum }}\ln\left(1-h_{l}^{\langle d\rangle }\right)+\delta_{l_{d}}^{\langle d\rangle }\ln h_{l_{d}}^{\langle d\rangle }\\ & \;\;\;+\left(1-\delta_{l_{d}}^{\langle d\rangle }\right)\ln\left(1-h_{l_{d}}^{\langle d\rangle }\right)\}. \end{array}$$

The unknown parameters **β** in (9) can thus be estimated by maximizing the partial log likelihood (12), which is the log likelihood for the independent Bernoulli trial. Although ln⁡*L*(**β**) is not a log likelihood in the usual sense, it possesses the usual asymptotic properties under fairly broad conditions; see, for example, Andelsen and Gill [26].

To avoid overfitting, such models are estimated by penalized maximum likelihood

(13) $$\begin{array}{cc} \ln L\left(\beta \right) & =\overset{n}{\underset{d=1}{\sum }}\{\overset{l_{d}-1}{\underset{l=1}{\sum }}\ln\left(1-h_{l}^{\langle d\rangle }\right)+\delta_{l_{d}}^{\langle d\rangle }\ln h_{l_{d}}^{\langle d\rangle }\\ & \;\;\;+\left(1-\delta_{l_{d}}^{\langle d\rangle }\right)\ln\left(1-h_{l_{d}}^{\langle d\rangle }\right)\}\\ & \;+\frac{1}{2}\overset{I}{\underset{i=1}{\sum }}\lambda_{i}\int {\left\{s_{i}^{\prime \prime }\left(t\right)\right\}}^{2}dt, \end{array}$$

where *λ*_*i*_ are smoothing parameters that control the tradeoff between the fit and the smoothness, and *s*_*i*_′′ is the twice derivative of *s*_*i*_ with respect to *t*. The advantage of penalized estimates is enlightened in Wood ([27], Section  4.1).

Two model-fitting issues remain. The first concerns the selection of smoothing parameter *λ*_*i*_ in (13). The careful smoothing parameter choice is outweighed by the easy identification of a covariate's functional form, and the applicability of established inferential methods to short-term survival prediction. In order to select the smoothing parameters, the algorithm due to Wood [27–29] can be used by minimizing GCV as an approximation to leaving-one-out CV. For example, however, the dead patient #9 generated seven observations as shown in Table 1. Patients were scheduled to return for further observations at six months, 12 months, and yearly. It should be noted that this patient generated seven observations. Thus, *n* = 312 patients generate 1945 observations in total.

We propose a natural extension of *v*-fold CV algorithm by “leaving-one-out” CV based on each *n* = 312 patients. The ordinal *v*-fold CV divides the data randomly in *v* groups so that their sizes are as nearly equal as possible. The partition should be made to avoid possible biases, as described in Zhang [30]. In many problems, the ordinal *v*-fold CV is, thus, unsatisfactory in several respects for time-dependent covariates. Applying this kind of data structure to the CV algorithm, we obtain insights into how the partition of data should be done. A natural extension of *v*-fold CV algorithm by setting *v* = *n* is to allow the deletion of the patient with several observations. The variant *v*-fold CV is given as follows:

**Step 1**

Split the original sample **X** = {**X** ^〈1〉^,…, **X** ^〈*d*〉^,…, **X** ^〈*n*〉^} into *n* parts **X** = {**X** ^〈1〉^ | …|**X** ^〈*d*〉^ | …|**X** ^〈*n*〉^}, where **X** ^〈*d*〉^ = {**X**_1_^〈*d*〉^,…, **X**_*l*_*d*__^〈*d*〉^}.

**Step 2**

Fit the model to X* = {**X** ^〈1〉^ | …|**X** ^〈*d*−1〉^|**X** ^〈*d*+1〉^ | …|**X** ^〈*n*〉^} with the *d*-th subject (i.e., patient) deleted of the data, and predict $\sum_{l=1}^{l_{d}-1}\ln\left(1-{\hat{h}}_{l}^{\langle d\rangle }\right)+\delta_{l_{d}}^{\langle d\rangle }\ln{\hat{h}}_{l_{d}}^{\langle d\rangle }+\left(1-\delta_{l_{d}}^{\langle d\rangle }\right)\ln\left(1-{\hat{h}}_{l_{d}}^{\langle d\rangle }\right)$ for the deleted *d*th sample **X** ^〈*d*〉^.

**Step 3**

Do the above for *d* = 1,2,…, *n* and combine the CV estimates

(14) $$\begin{array}{cc} \text{CV} & =\overset{n}{\underset{d=1}{\sum }}\{\overset{l_{d}-1}{\underset{l=1}{\sum }}\ln\left(1-{\hat{h}}_{l}^{\langle d\rangle }\right)+\delta_{l_{d}}^{\langle d\rangle }\ln{\hat{h}}_{l_{d}}^{\langle d\rangle }\\ & \;\;+\left(1-\delta_{l_{d}}^{\langle d\rangle }\right)\ln\left(1-{\hat{h}}_{l_{d}}^{\langle d\rangle }\right)\;\}. \end{array}$$

A second issue is the goodness-of-fit test of the model. After choosing the optimum smoothing parameters via *v*-fold CV algorithm, the deviance allows us to test the goodness of fit

(15) $$\text{Dev}=2\left(\ln L_{\max}-\ln L_{c}\right),$$

where ln⁡*L*_*c*_ denotes the maximized partial log likelihood under some current GAM, and the log likelihood for the maximum (full) model ln⁡*L*_max⁡_ is zero. The deviance given by (15) is, however, not even approximately a *χ* ^2^ distribution for the case in which ungrouped binary responses are available; see, for example, Landwehr et al. [31] and Tsujitani and Koshimizu [32] and Collett [33]. The number of degrees of freedom required for the test for significance using the assumed *χ* ^2^ distribution for the deviance is a contentious issue. No adequate distribution theory exists for the deviance. The reason for this is somewhat technical; for details, see Section  3.8 in Collett [33]. Consequently, the deviance on fitting a model to binary response data cannot be used as a summary measure of the goodness-of-fit test of the model.

Based on the above discussion, we employ bootstrapping to the deviance of (15) in order to obtain the goodness-of-fit test due to Efron and Tibshirani [34].

**Step 1**

Generate *B* bootstrap samples X* = {**X**_*l*_^〈1〉∗^,…, **X**_*l*_^〈*n*〉∗^} from the original sample **X**. Let X*(*b*) denote the *b*th bootstrap sample.

**Step 2**

For the bootstrap sample X*(*b*), the deviance of (15) is computed by $\text{Dev}\left(b\right)=2\left[\ln L_{f}-\ln\left(X^{*}\left(b\right);{\hat{\beta }}^{*}\left(b\right)\right)\right]$.

This process is repeated independently *B* times, and the computed values are arranged in ascending order.

**Step 3**

The value of the *j*th order statistic Dev* of the *B* replications can be taken as an estimate of the quantile of order *j*/(*B* + 1).

**Step 4**

The estimate of the 100(1 − *α*)-th percentile (i.e., *α*% critical point) of Dev* is used to test the goodness-of-fit of a model having a specified significance level *α* = 1 − *j*/(*B* + 1). The value of deviance of (15) being greater than the estimate of the percentile indicates that the model fits poorly.

## 3. Results

The survival function for our discretized situation is

(16) $$S\left(t_{l}\right)=\underset{1\leq i\leq l}{\prod }\left(1-h_{i}^{\langle d\rangle }\right).$$

The conditional probability Pr(*t*, *t* + Δ*t*) of survival over a short-time interval Δ*t* (say, six months) after time *t* can be estimated as

(17) $$\hat{\text{Pr}}\left(t,\Delta t\right)=\frac{\hat{S}\left(t+\Delta t\right)}{\hat{S}\left(t\right)}.$$

By using variant *v*-fold CV and GCV, the optimum smoothing parameters for GAM are determined as shown in Table 2.  Table 3 summarizes the *P* values to test the nonparametric effects of covariates for the model *s*(time) + *s*(age) + *s*(pro) + *s*(bili) with the optimum smoothing parameters and GCV. From Table 3, all covariates are highly significant for GCV; however, time is not significant for variant *v*-fold CV. GCV is only the approximation of leaving-one-out CV. Furthermore, the variant *v*-fold CV is leaving one-out CV based on each *n* = 312 patients. So variant *v*-fold CV is better than GCV. For the purpose of comparison, we included the results in the case using GCV in Tables 2 and 3. Table 4 shows the results of a number of models that were fit to the data.

The likelihood ratio (LR) statistic based on deviance can be conducted to test whether the spline effect provides a significantly better fit than a linear effect.  Table 5 shows the test of significance for spline effects based on the models in Table 4. It is clear from Table 5 that the spline effects of prothrombin time and bilirubin are strongly significant. No spline provides a significantly better fit than a linear model for age. Thus, we accept the final model: age + *s*(pro) + *s*(bili) with the edf(effective degrees of freedom) 3.954 and 4.477 for *s*(pro) and *s*(bili), respectively. We used likelihood ratio test instead of information criteria as a valid alternative approach for model selection. From Table 4, however, it is found that the same final model is selected by using AIC.

**Figure 1:**
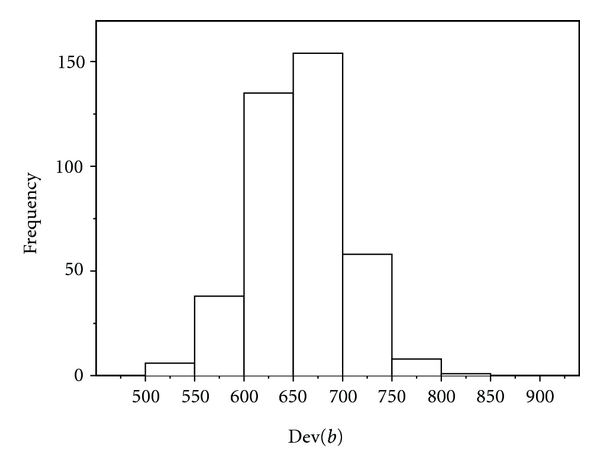
Histogram of the bootstrapped Dev(*b*) for *B* = 400.

Figure 1 shows the histogram of the bootstrapped Dev(*b*) for the optimum model with *B* = 400. The bootstrap estimate of the 95th percentile (i.e., 5% critical point) Dev* is Dev* = 734.59. Comparison to Dev = 663.65 of (15) suggests that the model fits the data.

**Figure 2:**
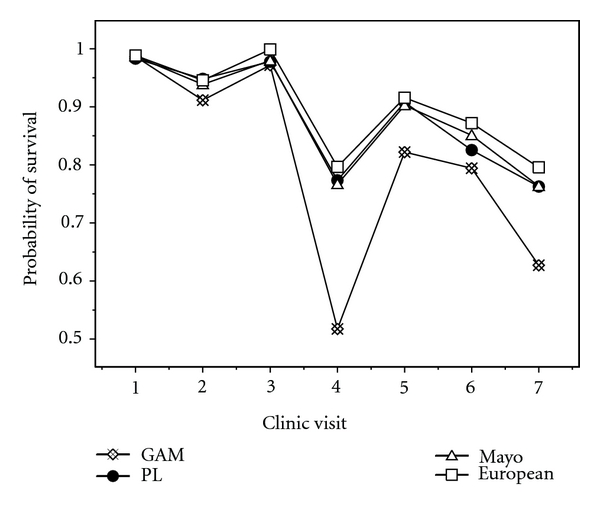
Probability of survival over the next six months using the four models with respect to dead patient #9.

Figure 2 shows the prediction of the probability of surviving beyond the next six months for dead patient #9. For the purpose of comparison, the results obtained using partial logistic regression, the Mayo updated, and the European new version models are also provided. Figure 2 also indicates that the six-month survival probability predicted by GAM are lower than those predicted by the other models. Because the patient #9 died, the lower predicted probabilities are better. The conditional probability Pr(*t*, Δ*t*) of survival over a short time interval Δ*t* (say, six months) after time *t* during the course of the disease can be predicted from data collected for censored and dead data.

For the graphical representation, the individual probabilities for predicted survival are averaged in order to compare the Mayo updated model, the European new version model, the partial logistic regression model, and GAM. We can predict the probability of survival over the following six months using the four models with respect to data and censored data out of 312 patients. For the group (*g* = 1) and the censored group (*g* = 2), the probability of surviving over the next Δ*t* months is denoted by Pr_*d*_^[*g*]^(*l*, *l* + Δ*t*) for the *l*-th clinic visit of patient #*d*. The average probability of survival over the next Δ*t* months for the *l*-th clinic visit of patient #*d* in group *g* can be estimated as

(18) $$\begin{array}{cc} S_{g}\left(l\right)=\frac{1}{n_{l}^{\left[g\right]}}\overset{n_{l}^{\left[g\right]}}{\underset{d=1}{\sum }}\text{P}{\text{r}}_{d}^{\left[g\right]}\left(l,l+\Delta t\right),\;g=1,2, & \\ l=1,2,\ldots ,L, & \end{array}$$

where *n*_*l*_^[*g*]^ is the total number of patients for the *l*-th clinic visit in group *g*, and Pr_*d*_^[*g*]^(*l*) is the survival function Pr(*l*) of patient #*d* at the time interval *l* in group *g*; see, for example, Markus et al. [35], Marubini and Valsecchi [10], and Thomsen et al. [36].

**Figure 3:**
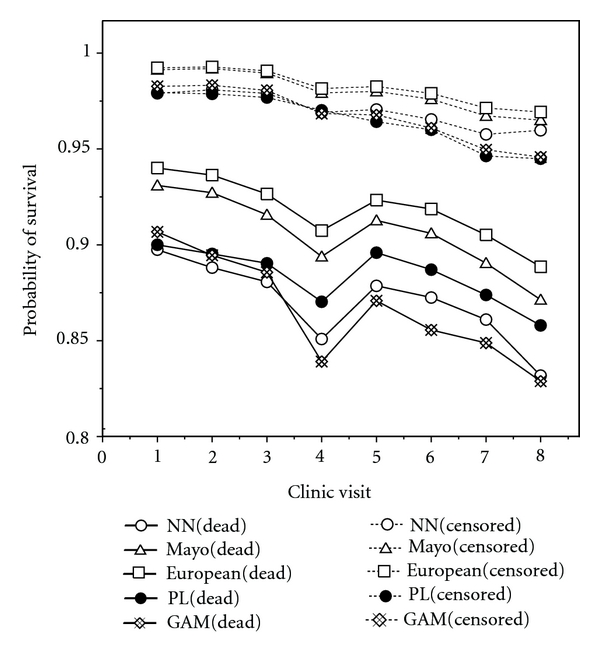
Probability of survival over the next six months using the four models with respect to dead and censored data among all 312 patients.

Figure 3 shows a comparison of the probability of survival over the next six months using the four models with respect to dead and censored data among all 312 patients. The figure clarifies that

- for the case of dead data, the six-month survival probabilities predicted by GAM are lower than those predicted by the other models, and,
- for the case of censored data, the difference among the four models is very small.

**Figure 4:**
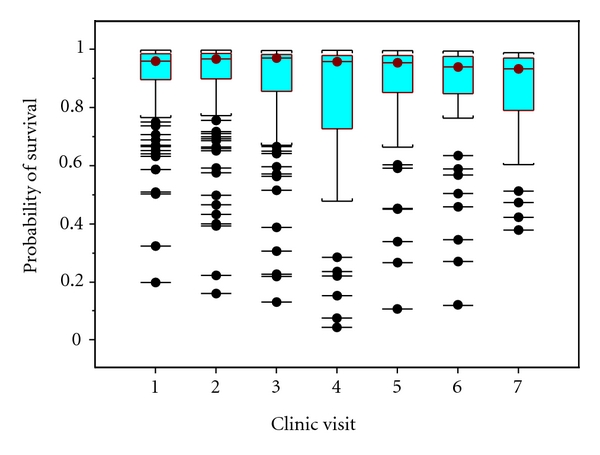
Box and whisker plots of probability of survival over the next six months using GAM with respect to dead data among all 312 patients.

Figure 4 also shows the box and whisker plots of probability of survival over the next six months using GAM with respect to dead data among all 312 patients. It should be noted that the variance of probabilities of survival over the next six months is much higher in the fourth clinic visits than in other clinic visits. Another useful idea in our analysis is the concept of competing risk. There is “liver transplantation” in PBC data as competing risk. Competing risk has been treated as censored data. By adding *x*_*l*4_^〈*d*〉^ as one of time-dependent covariate for the liver transplantation, one can test the significance of liver transplantation. The covariate for liver transplantation is taken as a binary variable (codes 0 before liver transplantation, 1 at liver transplantation) as shown in Giorgi and Gouvernet [16] and Crowley [37].  Table 6 shows the three types for the combination of “censored” and “liver transplantation.” Table 7 shows the values of covariates for liver-transplanted patient #5.

In order to test the significance of “liver transplantation,” we consider two models:

Model IV: age + *s*(pro) + *s*(bili).

Model IV′: age + *s*(pro) + *s*(bili) + liver transplantation. The values of deviance and d.f. are given in Table 8. The reduction in the value of deviance is 10.657 = 663.65 − 653.00 on 0.973 d.f. This is significant at the 1% level.

For the purpose of the comparison, the hazard of the cumulative incidence function (CIF) may be modeled in the presence of competing risks. The model is based on

(19) $$\gamma \left(t;x\right)=\gamma_{0}\left(t\right)\exp\left[\overset{I}{\underset{i=1}{\sum }}\beta_{i}x_{i}\right],$$

where *t* is the time of the last observation (not the midpoint at the time interval *l*), *γ* is the hazard of the subdistribution, and *γ*_0_ is the baseline hazard of the subdistribution ([38], Section  6.2). The *P* values are summarized in Table 8 to test the significance for covariates using the model (19). From Tables 3 and 8, there is little difference between our method and the CIF.

## 4. Discussion

In this paper, we introduced the probabilistic interpretation of GAM and constructed the maximum likelihood principle of GAM for the analysis of survival data having time-dependent covariates. We proposed the information criterion based on the variant *v*-fold CV when choosing the optimal smoothing parameters in application of GAM. Introducing the maximum likelihood principle into GAM, the deviance allows us to test the goodness-of-fit of GAM. The proposed methods were illustrated by comparing the probability of survival over the next six months using the Mayo-updated model, the European new version model, the partial logistic regression model, and GAM with respect to dead and censored data among PBC data. We expect that flexible methods for modeling survival data with time-dependent covariates using machine learning theory such that support vector machine will be very useful in this real-world contexts; see, for example, Hastie et al. [39]. Furthermore, smoothing spline ANOVA models by Gu [40] will enable us to include the interactions between the covariates.

We assume that there is only one cause of failure; that is, the event is allowed to occur only once for each patient. However, there is increasing interest to apply survival data sets with multiple events per patient [8, 41]. Wei et al. [42] analyzed bladder cancer data by modeling marginal distributions of multivariate failure time with proportional hazards models. The model may violate the proportional hazards assumption, even when the overall data set does not (Table 9). By modifying such as

(20) $$\delta_{l}^{\langle d\rangle }=\{\begin{array}{cc} \text{1}\;\;\text{:}\;\;\text{recurrent event for patient}\;\;\#d & \\ \text{ occurred at the time-interval}\;\;l & \\ 0:\text{otherwise.} & \end{array}$$

Equation (11) becomes

(21) $$\begin{array}{cc} \ln L\left(\beta \right) & =\overset{n}{\underset{d=1}{\sum }}\;\overset{l_{d}}{\underset{l=1}{\sum }}\left\{\delta_{l_{d}}^{\langle d\rangle }\ln h_{l_{d}}^{\langle d\rangle }+\left(1-\delta_{l_{d}}^{\langle d\rangle }\right)\ln\left(1-h_{l_{d}}^{\langle d\rangle }\right)\right\} \end{array}.$$

Thus, the ideas presented in this paper can be extended to identification of prognostic factors relative to survival time in the case that the same event may recur during a follow-up study, and covariates values change with time.

## Figures and Tables

**Table 1:** Values of covariates for deceased patient #9.

Time interval *l*	Midpoint day *a*_*l*_^〈9〉^	Age (year) *x*_*l*1_^〈9〉^	Prothrombin time (sec) *x*_*l*2_^〈9〉^	Bilirubin (mg/dL) *x*_*l*3_^〈9〉^	*δ*_*l*_^〈9〉^
1	92.0	42.54	11.0	3.2	0
2	272.5	43.04	12.5	7.0	0
3	542.0	43.53	11.2	4.2	0
4	875.0	44.52	14.1	13.5	0
5	1211.5	45.35	11.5	12.0	0
6	1837.0	46.36	11.5	16.2	0
7	2339.0	48.78	13.0	14.8	1

**Table 2:** Optimum smoothing parameters.

Covariates	Variant *v*-fold CV	GCV
Time	0.1	0.00045
Age	10	0.000015
Prothrombin time	0.001	0.00039
Bilirubin	0.001	0.00000017

**Table 3:** Test of significance for the covariates (*P* values).

Covariates	Variant *v*-fold CV	GCV
Time	0.263	0.0028
Age	<0.0001	<0.0001
Prothrombin time	<0.0001	<0.0001
Bilirubin	<0.0001	<0.0001

**Table 4:** Ranks and *F* values for nonparametric effects.

No.	Model	Deviance	d.f.	CV (AIC)
I	*s*(time) + *s*(age) + *s*(pro) + *s*(bili)	661.77	1933.16	691.99 (685.44)
II	*s*(age) + *s*(pro) + *s*(bili)	663.65	1934.57	690.54 (684.52)
III	*s*(time) + *s*(pro) + *s*(bili)	705.50	1933.93	739.03 (727.64)
IV	Age + *s*(pro) + *s*(bili)	663.65	1934.57	690.51 (684.51)
V	Age + *s*(pro) + bili	686.63	1937.58	708.44 (701.46)
VI	Age + pro + *s*(bili)	675.80	1938.02	696.53 (689.77)

**Table 5:** Test of significance for spline effects.

Spline effect	Δ	d.f.
Age	0.0039 (Model IV-II)	0.005
Prothrombin time	12.15 (Model VI-IV)	3.45
Bilirubin	22.97 (Model V-IV)	3.01

**Table 6:** Three types of “censored” and “liver transplantation.”

Dead patient		Censored patient		Liver-transplanted patient	
*δ*_*l*_^〈*d*〉^	Liver transplant	*δ*_*l*_^〈*d*〉^	Liver transplant	*δ*_*l*_^〈*d*〉^	Liver transplant
0	0	0	0	0	0
.	.	.	.	.	.
.	.	.	.	.	.
1	0	0	0	0	1

**Table 7:** Values of covariates for dead patient #5.

Time interval *l*	Midpoint day *a*_*l*_^〈9〉^	Age (year) *x*_*l*1_^〈9〉^	Prothrombin time (sec) *x*_*l*2_^〈9〉^	Bilirubin (mg/dL) *x*_*l*3_^〈9〉^	Liver trans-plantation *x*_*l*4_^〈9〉^
1	99.5	38.11	10.9	3.4	0
2	295.0	38.65	10.7	1.9	0
3	580.0	39.18	10.5	2.5	0
4	933.5	40.21	11.4	5.7	0
5	1276.5	41.11	11.3	5.2	0
6	1480.0	42.09	13.9	19.0	1

**Table 8:** Test of significance for covariates using model (19).

Covariate	*P*-value
Age	<0.0001
Prothrombin time	<0.0001
Bilirubin	<0.0001

**Table 9:** Deviance and d.f.

Model	Deviance	d.f.
IV	663.65	1934.57
IV′	653.00	1934.00
